# Characterisation of a 3-hydroxypropionic acid-inducible system from *Pseudomonas putida* for orthogonal gene expression control in *Escherichia coli* and *Cupriavidus necator*

**DOI:** 10.1038/s41598-017-01850-w

**Published:** 2017-05-11

**Authors:** Erik K. R. Hanko, Nigel P. Minton, Naglis Malys

**Affiliations:** BBSRC/EPSRC Synthetic Biology Research Centre (SBRC), School of Life Sciences, Centre for Biomolecular Sciences, The University of Nottingham, Nottingham, NG7 2RD United Kingdom

## Abstract

3-hydroxypropionic acid (3-HP) is an important platform chemical used as a precursor for production of added-value compounds such as acrylic acid. Metabolically engineered yeast, *Escherichia coli*, cyanobacteria and other microorganisms have been developed for the biosynthesis of 3-HP. Attempts to overproduce this compound in recombinant *Pseudomonas denitrificans* revealed that 3-HP is consumed by this microorganism using the catabolic enzymes encoded by genes *hpdH*, *hbdH* and *mmsA*. 3-HP-inducible systems controlling the expression of these genes have been predicted in proteobacteria and actinobacteria. In this study, we identify and characterise 3-HP-inducible promoters and their corresponding LysR-type transcriptional regulators from *Pseudomonas putida* KT2440. A newly-developed modular reporter system proved possible to demonstrate that *Pp*MmsR/P_*mmsA*_ and *Pp*HpdR/P_*hpdH*_ are orthogonal and highly inducible by 3-HP in *E. coli* (12.3- and 23.3-fold, respectively) and *Cupriavidus necator* (51.5- and 516.6-fold, respectively). Bioinformatics and mutagenesis analyses revealed a conserved 40-nucleotide sequence in the *hpdH* promoter, which plays a key role in HpdR-mediated transcription activation. We investigate the kinetics and dynamics of the *Pp*HpdR/P_*hpdH*_ switchable system in response to 3-HP and show that it is also induced by both enantiomers of 3-hydroxybutyrate. These findings pave the way for use of the 3-HP-inducible system in synthetic biology and biotechnology applications.

## Introduction

Chemicals and fuels can be produced from renewable or waste feedstocks using metabolically engineered microorganisms, which may aid to reducing greenhouse gas emissions^1–3^. Recently, significant research efforts have been directed towards developing microorganisms for the biosynthesis of value-added chemicals, including 3-hydroxypropionic acid (3-HP)^4–7^. 3-HP is a biotechnologically attractive platform chemical that can be used as a precursor for biosynthesis of acetaldehyde, acrylate, acrylamide, methylacrylate, and 1,3-propanediol^6^, as well as biodegradable polymer poly-3-HP^8^. A number of recombinant strains using *Corynebacterium glutamicum, Escherichia coli, Klebsiella pneumoniae, Lactobacillus reuteri, Pseudomonas denitrificans, Synechocystis sp., Synechococcus elongatus* and *Saccharomyces cerevisiae* as chassis^9–16^, and a few alternative metabolic pathways have been developed for 3-HP biosynthesis using intermediates such as β-alanine, malonyl-CoA, propionyl-CoA, glycerol and lactate^17–21^. Although, relatively high titres of 3-HP have been reported in *E. coli* and *K. pneumoniae* ^18, 22^, the challenge remains to develop a sustainable biotechnological production of this carboxylic acid^23^.

3-HP can be efficiently assimilated and utilised as a carbon and energy source by bacteria. *P. denitrificans* possesses 3-HP dehydrogenase and 3-hydroxyisobutyrate dehydrogenase activities which contribute to 3-HP degradation^10, 24^. The expression of some genes related to 3-HP metabolism in *P. denitrificans* have been shown to be strongly induced by 3-HP and putatively controlled by transcriptional regulators (TR)^24^, which belong to the family of LysR-type TRs (LTTRs)^25^.

LTTRs contain a conserved protein structure with a DNA binding helix-turn-helix motif at the N-terminus and an effector binding domain at the C-terminus. They are typically activated by small effector molecules and regulate the transcription of metabolism-related genes. Most often, LTTRs are encoded in opposite direction of the gene or operon that they regulate. They usually interact with at least two TR binding sites in the intergenic region^25, 26^. The regulatory binding site is located 60–80 nucleotides upstream with respect to the transcriptional start site of the ligand-metabolizing gene. This motif has been characterised as T-N_11_-A, which can vary in length and/or both nucleotide composition. Frequently, this site overlaps with the promoter of the LTTR gene mediating negative autoregulation. The activator binding site is usually adjacent or overlaps with the −35 box of the promoter^25^. Besides, examples with a single and multiple LTTR-binding sites have also been reported^27, 28^. Based on full length and domain analysis, in *Pseudomonas* LTTRs can be dissected into 9 evolutionary different phylogenetic groups^29^.

LTTR- and other TR-based switchable (inducible or repressible) systems regulate microbial gene expression in response to the change of intracellular levels of metabolites and play an important role in governing metabolic pathways and networks. Recently, a number of such switchable systems have been adapted as genetically-encoded biosensors and reporters that respond to a variety of native and non-native compounds^30–34^. Both the native and synthetic systems, in combination with a reporter gene, have been used to screen for metabolically engineered microbial strains enabling selection for microorganisms with improved production of target compounds^35–38^. They have also been applied as metabolite-responsive gene switches and dynamic regulators of metabolic pathways, in which levels of upstream or downstream gene expression are continuously adjusted to balance metabolic intermediate levels increasing the flux towards the product of interest^37, 39^.

*Pseudomonas* are an attractive source for exploration of novel gene targets such as TR-based switchable systems. Particularly, *Pseudomonas putida* KT2440 exhibits combinations of features characteristic to aquatic oligotrophs and terrestrial copiotrophs indicating that this bacterium has adapted functional capabilities permitting to thrive in various environments^40^. This genetically diverse microorganism contains a high affinity nutrient acquisition and metabolite efflux, as well as catabolic enzymes such as mono- and di-oxygenases, oxidoreductases and dehydrogenases. Additionally, *P. putida* also possesses a wide range of gene expression control systems involving various sigma factors and regulators which form a rich genomic basis for the exceptional metabolism versatility.

Here, we report the identification and characterisation of a 3-HP-inducible system from *P. putida*, which is composed of a LTTR and a corresponding 3-HP-responsive promoter. As demonstrated in this study, it can be used to control gene expression orthogonally in *E. coli*, a model gammaproteobacterium, and *Cupriavidus necator*, a chemolithoautotrophic betaproteobacterium with the potential to produce chemicals from carbon dioxide. A comprehensive analysis of the promoter region is performed to establish a consensus sequence required for potential TR binding. The characterised switchable system can be exploited as 3-HP biosensor, as it shows a high specificity and a wide induction range for this compound, as well as genetic element for the construction of autoregulated metabolic pathways aiming to improve the production of bio-based 3-HP.

## Results

### Identification of 3-hydroxypropionic acid-inducible systems in *P. putida* KT2440 and *C. necator* H16

**Figure 1:**
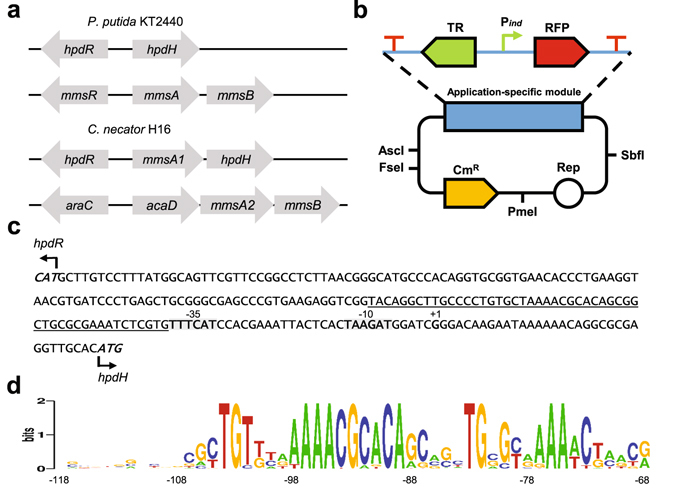
Identification of putative 3-HP-responsive genes in *P. putida* KT2440 and *C. necator* H16. (**a**) Operons putatively involved in 3-HP metabolism in *P. putida* KT2440 and *C. necator* H16. They are composed of the TR coding sequences *hpdR*, *mmsR* or *araC*, and divergently transcribed genes which are putatively required for 3-HP degradation: *hpdH*, 3-hydroxypropionate dehydrogenase; *mmsA*, methylmalonate-semialdehyde dehydrogenase; *mmsB*, 3-hydroxyisobutyrate dehydrogenase and *acaD*, acyl-CoA dehydrogenase. (**b**) Schematic illustration of the modular reporter system. It contains the four unique restriction sites AscI, FseI, PmeI and SbfI which are used for modular assembly. The application-specific module harbours the *rfp* reporter gene and the switchable system composed of transcriptional regulator and inducible promoter. (**c**) The *P. putida* KT2440 *hpdR*/*hpdH* intergenic region. Translational start sites are bold and italicised. The predicted *hpdH* −35 and −10 regions are bold and highlighted in grey. The nucleotide sequence between position −118 and −68 relative to the *P. putida* KT2440 *hpdH* translational start site is underlined. (**d**) A sequence similarity motif was generated which corresponds to the underlined sequence. It represents highly conserved nucleotides in the *hpdR*/*hpdH* intergenic regions of forty analysed *Pseudomonas* species.

Three enzymes have been identified in *P. denitrificans* to be involved in 3-HP degradation^24^. They are arranged in two operons. The 3-hydroxypropionate dehydrogenase (*hpdH*) in one operon and the methylmalonate-semialdehyde dehydrogenase (*mmsA*) and 3-hydroxyisobutyrate dehydrogenase (*mmsB*, also referred to as *hbdH*-4) in the second operon. In the opposite direction of each operon, a gene encoding a LTTR is located which was proposed to be required for 3-HP-inducible activation of gene transcription of its respective operon^24^. Homologues of the 3-HP catabolic genes have been found in various microbial genera including *Cupriavidus* ^24^. In this study we identified HpdH, MmsA and MmsB homologues in the genomes of *P. putida* KT2440 and *C. necator* H16 (Supplementary Table S1). In *P. putida*, the 3-HP catabolic genes and their respective LTTRs are as arranged as in *P. denitrificans* (Fig. 1a). In *C. necator*, however, the arrangement of genes is different. A *P. denitrificans* MmsA homolog with 49% protein sequence identity and 96% coverage is located between the *C. necator hpdH* and its putative LTTR (here termed HpdR). The short intergenic region of 27 bp between the *mmsA* homolog (here termed *mmsA1*) and *hpdH* suggests an operonic transcription of genes from a promoter located in the intergenic region between *mmsA1* and *hpdR*. For *mmsA* (here termed *mmsA2*) and *mmsB*, both arranged in one operon, no LTTR was found upstream of the genes. An acyl-CoA dehydrogenase, *acaD*, is annotated upstream of *mmsA2*. Both genes are oriented in the same direction. They are separated only by a short intergenic region of 44 bp which suggests an operonic arrangement of genes. In the opposite orientation of *acaD*, a transcriptional regulator is located which is annotated to belong to the AraC family. The regulator does not share protein sequence similarity with the *P. denitrificans* LTTR, which is putatively required to activate transcription of the *mmsAB* operon. However, it was included in the analysis for 3-HP-inducible gene expression as potential regulator of the *C. necator acaD*-*mmsA2-mmsB* operon.

### Construction of a modular reporter system for evaluation of switchable systems

In order to determine the ability of different putative switchable systems to mediate gene expression in response to 3-HP, a modular reporter system was constructed (Fig. 1b). The reporter plasmid comprises the following features: (i) a pBBR1MCS derived broad-host-range vector replicon^41^, which allows for replication in Gram-negative bacteria, (ii) compatibility with the pMTL vector series enabling rapid exchange of replication origin and selection marker^42^, (iii) various restriction sites within the application-specific module to facilitate simple replacement of switchable system and *rfp* reporter gene^43^, and (iv) transcriptional terminators flanking the application-specific module to prevent background reporter gene expression. The reporter plasmid which has been employed as origin for all other constructs, pEH006, contains the arabinose-inducible system. It was used to validate the modular reporter construct and to make our results comparable to previously reported data. As a control, an identical plasmid lacking both the TR AraC and the arabinose-inducible promoter, pEH006E, was assembled. Single time point fluorescence measurements were performed in *E. coli* MG1655 and *C. necator* H16 harbouring the reporter plasmid. The arabinose-inducible system was tested and demonstrated more than a 1,200-fold increase of RFP expression in *C. necator* after six-hour induction with 0.1% (w/v) L-arabinose. This level of induction is significantly higher than what has been previously reported for a similar construct^44^. Fluorescence of cells containing pEH006E was similar to the background fluorescence of the medium, indicating no transcriptional read-though from the vector backbone. Therefore, its insulating backbone makes pEH006 a suitable original modular vector for construction of switchable systems.

### *P. putida* KT2440 3-HP-inducible systems outperform systems derived from *C. necator* H16

Following the validation of the reporter construct, the putative 3-HP-inducible gene expression systems were cloned upstream of *rfp* into pEH006. They contain the TR and the intergenic region between the regulator coding sequence and the translational start site of the operons of the 3-HP catabolic genes. The two systems from *P. putida* KT2440 are referred to as *Pp*MmsR/P_*mmsA*_ (pEH007) and *Pp*HpdR/P_*hpdH*_ (pEH008) and those from *C. necator* H16 as *Cn*AraC/P_*acaD*_ (pEH009) and *Cn*HpdR/P_*mmsA1*_ (pEH010). The constructs were tested in *E. coli* MG1655 and *C. necator* H16 for *rfp* reporter gene expression in response to 3-HP. Both 3-HP-inducible systems from *P. putida* exhibit statistically significant induction (p < 0.01) in *E. coli* and *C. necator* six hours after addition of 10 mM 3-HP to the culture (Table 1). *Pp*HpdR/P_*hpdH*_ demonstrates the highest level of normalized fluorescence and the strongest induction values; 23.3-fold in *E. coli* and more than 500-fold in *C. necator*. In contrast to *C. necator* in which *Cn*HpdR/P_*mmsA1*_ shows a 88.4-fold induction, neither of the transcriptional regulators derived from *Cupriavidus* seem to be able to activate reporter gene expression from their proposed cognate promoters in *E. coli*. Besides, *Cn*AraC/P_*acaD*_ does not mediate statistically significant (p < 0.01) reporter gene expression after addition of 10 mM 3-HP in *C. necator*.

**Table 1:** Comparison of normalized fluorescence of cells carrying the different versions of putative 3-HP-inducible gene expression systems from *P. putida* KT2440 (*Pp*) and *C. necator* H16 (*Cn*) in the presence or absence of 10 mM 3-HP.

Switchable system	*E. coli* MG1655			*C. necator* H16		
	Fluorescence intensity/OD_600_		Induction ratio	Fluorescence intensity/OD_600_		Induction ratio
	Uninduced	Induced		Uninduced	Induced	
*Pp*MmsR/P_*mmsA*_	59 ± 3	721 ± 24	12.3*	444 ± 80	22,857 ± 1808	51.5*
*Pp*HpdR/P_*hpdH*_	1,259 ± 68	29,351 ± 756	23.3*	304 ± 46	157,052 ± 8,409	516.6*
*Cn*AraC/P_*acaD*_	0 ± 0	0 ± 0	0	311 ± 16	464 ± 69	1.5
*Cn*HpdR/P_*mmsA1*_	22 ± 6	26 ± 5	1.2	847 ± 27	74,839 ± 1,763	88.4*
*Pp*P_*mmsA*_	193 ± 16	243 ± 23	1.3	44 ± 8	42 ± 4	1.0
*Pp*P_*hpdH*_	908 ± 60	1,077 ± 47	1.2	42 ± 1	40 ± 3	1.0

Each system is composed of either a putative 3-HP-inducible promoter and its respective transcriptional regulator or a 3-HP-inducible promoter only. The mean values and standard deviations represent the normalized fluorescence of biological triplicates six hours after induction for *E. coli* MG1655 and *C. necator* H16. Asterisks indicate statistically significant induction values for p < 0.01 (unpaired *t* test).

### The *Pp*MmsR/P_*mmsA*_ and *Pp*HpdR/P_*hpdH*_ switchable systems demonstrate a TR-dependent orthogonality in *E. coli* MG1655 and *C. necator* H16

Ideal TR-based switchable gene expression systems are specific to a certain metabolite or exogenously added inducer and are controlled by its corresponding TR only. In order to examine the cross-reactivity of host-originating transcription factors on reporter gene expression, the regulator coding sequences were removed from the plasmids that initially contained the *Pp*MmsR/P_*mmsA*_ and *Pp*HpdR/P_*hpdH*_ switchable systems. Constructs are solely composed of the 3-HP-inducible promoters P_*mmsA*_, or P_*hpdH*_ upstream of the *rfp* reporter gene. *Cn*HpdR/P_*mmsA1*_ and *Cn*AraC/P_*acaD*_ were not further investigated since they demonstrated no induction of reporter gene expression upon supplementation of 3-HP in *E. coli* and only in case of *Cn*HpdR/P_*mmsA1*_ a statistically significant (p < 0.01) induction in *C. necator*.

In both *E. coli* and *C. necator*, neither of the solely *P. putida* derived promoters demonstrated statistically significant (p < 0.01) induction of RFP expression after addition of 3-HP (Table 1). This shows that their corresponding LTTRs are required for transcription activation and that *E. coli* and *C. necator* do not have cross-reacting regulator homologues. Since P_*hpdH*_ demonstrated the highest induction level of the analysed 3-HP-inducible systems, and is controlled independently from host-originating TRs in *E. coli* and *C. necator*, it was chosen to be further characterised.

### Identification of a conserved sequence motif within the *hpdH* promoter and *in vivo* analysis of the HpdR binding site

Control of gene expression in response to transcription-inducing metabolites (TIMs) is mainly mediated by TRs. They interact with conserved *cis*-acting regulatory elements (CRE) in the promoters of genes which are involved in TIM-converting pathways. A simple approach to identify CREs is to compare sequences from a variety of divergent species^45^. By multiple sequence alignment, conserved functional elements can easily be distinguished from sequences that are prone to evolve more quickly.

*P. putida* KT2440 HpdR and HpdH homologues were screened in other *Pseudomonas* species by searching the NCBI database for non-redundant protein sequences. Forty different *Pseudomonas* species were selected with HpdR and HpdH protein sequence identities of at least 70% (Supplementary Table S2). To aid in the identification of the putative HpdR binding site, the consensus sequence was determined in the intergenic region as described in *Methods*. The *P. putida* KT2440 *hpdR*/*hpdH* intergenic region is illustrated in Fig. 1c. Upstream of the *hpdH* −35 region, a range of nucleotides was found which are highly conserved across the analysed *Pseudomonas* species. This region is illustrated as a sequence similarity motif in Fig. 1d. It corresponds to the nucleotide sequence between position −118 and −68 relative to the *P. putida* KT2440 *hpdH* translational start site. Two partially similar and conserved sequence motifs can be identified. The first is an imperfect inverted repeat, sequence GCCCCTGTGC-n_6_-GCACAGCGGC in *P. putida*, located between positions −109 and −84 and is referred to as *cis*-acting regulatory element 1 (CRE1). The second motif is located between −83 and −68 and denoted as CRE2. In order to test if the highly conserved nucleotides in −109 to −68 are essential for promoter activation mediated by HpdR in response to 3-HP, the *P. putida* KT2440 *hpdR*/*hpd*H intergenic region was truncated and subsequently mutated.

**Figure 2:**
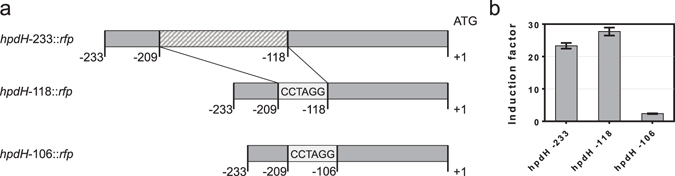
*P. putida* KT2440 *hpdH* promoter truncations. (**a**) Schematic illustration of the different *hpdH* promoter truncations that were evaluated for 3-HP-responsive activation of *rfp* reporter gene expression. The native promoter-reporter gene construct is denoted *hpdH*-233::*rfp*. The bioinformatically identified palindromic sequence is illustrated as a dashed box. This sequence was replaced by an AvrII restriction site, CCTAGG, resulting in promoter truncations *hpdH*-118::*rfp* and *hpdH*-106::*rfp*. (**b**) Induction levels mediated by the different promoter truncations. The various promoter-reporter gene constructs were analysed in *E. coli* MG1655 for RFP expression in the absence or presence of 10 mM 3-HP. Error bars represent standard deviations of three biological replicates.

Firstly, the −233 bp long intergenic region was analysed for secondary structures using the mfold web server^46^. A palindromic sequence was identified which can form a 97 bp spanning mRNA stem-loop structure (Supplementary Fig. S1). Interestingly, from the forty selected *Pseudomonas* species, this secondary structure is only present in *P. putida* KT2440 and the closely related *Pseudomonas entomophila* L48 and *Pseudomonas plecoglossicida* NBRC 103162. Likely, it has a species specific functional role, but is not evolutionary conserved. The reporter gene construct harbouring the native *hpdR*/*hpdH* intergenic region is referred to *hpdH*-233::*rfp*. The promoter truncation *hpdH*-118::*rfp* was generated by removing this palindromic sequence and incorporating an AvrII restriction site to allow further promoter modification (Fig. 2a). It contains the 118 bp long sequence upstream of the *hpdH* translational start site. Using *hpdH*-118::*rfp*, the promoter was truncated by additional twelve nucleotides, resulting in *hpdH*-106::*rfp*. The promoter-reporter gene constructs were analysed in *E. coli* MG1655 for their response to 3-HP (Fig. 2b). Compared to the native intergenic region (*hpdH*-233::*rfp*), the induction level slightly increased when the palindromic sequence was removed (*hpdH*-118::*rfp*). However, further truncation of the *hpdH* promoter by 12 nucleotides (*hpdH*-106::*rfp*) results in a 10-fold decrease of induction compared to that of *hpdH*-233::*rfp*.

**Figure 3:**
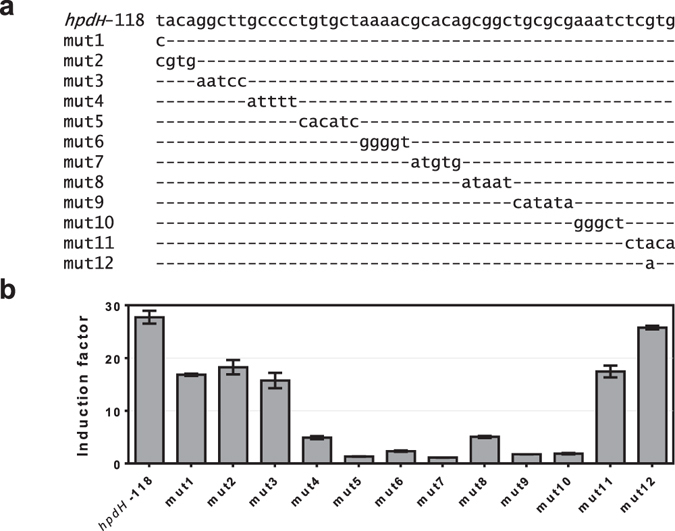
*P. putida* KT2440 *hpdH*-118::*rfp* promoter mutations. (**a**) Schematic illustration of the different *hpdH*-118::*rfp* promoter mutations that were evaluated for 3-HP-responsive activation of *rfp* reporter gene expression. Unchanged nucleotides are represented as dash. (**b**) Induction levels mediated by the different promoter mutations. The various promoter-reporter gene constructs were analysed in *E. coli* MG1655 for RFP expression in the absence or presence of 10 mM 3-HP. Error bars represent standard deviations of three biological replicates.

Since *hpdH*-118::*rfp* demonstrates similar induction levels to the native, untruncated intergenic region, it was subsequently used to generate *hpdH* promoter mutants. The use of random mutagenesis was deemed to be a combinatorial challenge, seeking to achieve a full set of completely random mutants covering the 60 bp long conserved motif. Therefore, mutations were designed in such a way that they enabled a complete coverage by employing a mutagenesis strategy, which has been reported previously^47^. In total, twelve promoter variants were constructed, harbouring single or multiple nucleotide mutations between position −118 and −68 (Fig. 3a). They were analysed in *E. coli* MG1655 for reporter gene expression in the presence and absence of 3-HP (Fig. 3b). By comparing the sequence similarity motif (Fig. 1d) with the induction levels of the different promoter variants, it can be observed that the extent of nucleotide conservation correlates with their importance for inducible gene expression. Mutations 1, 2, and 3, which are upstream of the −109 - −68 region, show a minor impact on promoter activity (1.5- to 1.7-fold decrease in induction) compared to *hpdH*-118::*rfp*. Mutations 4, 8 and 11, which are located in less conserved stretches of the −109 - −68 region, decrease induction of reporter gene expression by 5.5-, 5.4- and 1.6-fold, respectively. The remaining five mutations, 5, 6, 7, 9 and 10 alter the most conserved nucleotides in either CRE1 or CRE2. These mutations abolish the ability of the promoter to mediate controllable gene expression by decreasing induction levels from 11.5- (mutation 6) to 25.2-fold (mutation 7). The *in vivo* analysis of various *hpdH* promoter truncations and mutations suggests that a 50 bp long sequence upstream of the −35 region is involved in 3-HP-inducible gene expression.

Finally, to confirm that HpdR binds directly to the *hpdH* promoter region, the TR was purified and electrophoretic mobility shift assay (EMSA) was performed as described in *Supplementary Methods*. A DNA shift can clearly be observed even in the absence of 3-HP (Supplementary Fig. S2), suggesting that regulator binding to the intergenic region occurs even under uninduced conditions. Addition of 3-HP to the binding reaction on the other hand results in formation of a slower migrating complex 2. A similar observation was made for *catBC* promoter, where a tighter and potentially higher-order TIM-protein-DNA complex was proposed to facilitate activation of gene expression^48^.

### Determination of inducer-dependent orthogonality of the *Pp*HpdR/P_*hpdH*_ switchable system in *E. coli* MG1655 and *C. necator* H16

**Figure 4:**
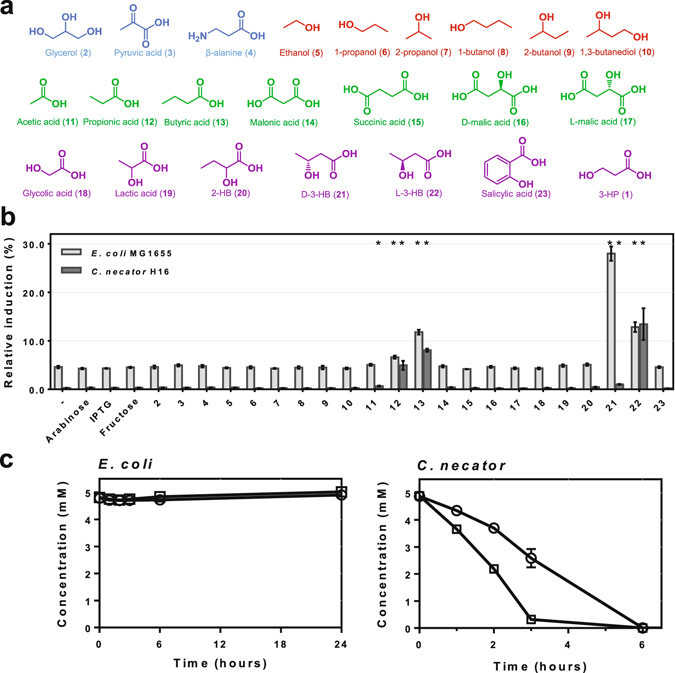
Determination of TIM-dependent orthogonality. (**a**) Compounds that were investigated for cross-reactivity with the *Pp*HpdR/P_*hpdH*_ switchable system: 3-hydroxypropionic acid (1), glycerol (2), pyruvic acid (3), β-alanine (4), ethanol (5), 1-propanol (6), 2-propanol (7), 1-butanol (8), 2-butanol (9), 1,3-butanediol (10), acetic acid (11), propionic acid (12), butyric acid (13), malonic acid (14), succinic acid (15), D-malic acid (16), L-malic acid (17), glycolic acid (18), lactic acid (19), 2-hydroxybutyric acid (20), D-3-hydroxybutyric acid (21), L-3-hydroxybutyric acid (22) and salicylic acid (23). (**b**) Relative induction levels of the *Pp*HpdR/P_*hpdH*_ switchable system which was subjected to a variety of putative TIMs. The various compounds were tested in *E. coli* MG1655 (light grey) and *C. necator* H16 (dark grey) harbouring pEH008. Error bars represent standard deviations of three biological replicates. Asterisks indicate statistically significant induction values for p < 0.01 (unpaired *t* test). (**c**) Consumption of D- (21, square) and L-3-HB (22, circle) in *E. coli* MG1655 and *C. necator* H16. Error bars represent standard deviations of three biological replicates.

In addition to host-originating TRs, which may interfere with the heterologous switchable system, other metabolites than the primary inducer may be able to activate gene expression. These can be either compounds that are involved in the cells metabolism, constituents of the growth medium or additional TIMs that were supplemented to control independent gene expression from other switchable promoters. Compounds that were investigated for cross-reactivity with the *Pp*HpdR/P_*hpdH*_ switchable system (Fig. 4a) include: (i) the commonly used inducers L-arabinose and IPTG, (ii) fructose as alternative carbon source, (iii) the 3-HP precursors glycerol (**2**), pyruvate (**3**) and β-alanine (**4**) (shown in blue)^49^, and (iv) a broad range of compounds that are structurally similar to 3-HP such as mono- and dihydric alcohols (shown in red), mono- and dicarboxylic acids (shown in green), as well as α- and β-hydroxycarboxylic acids (shown in purple). Evaluation of these compounds may aid in the determination of the structural features of the ligand which are required to interact with the TR. Additionally, it may assist to identify an analogue of 3-HP as inducer if 3-HP is metabolized as shown in *P. denitrificans* ^10^. In order to achieve a sustained gene expression from the *hpdH* promoter, either 3-HP metabolizing genes need to be deleted or a metabolically inert analogue inducer should be employed.

Single time point fluorescence measurements were performed in *E. coli* MG1655 and *C. necator* H16 carrying the reporter plasmid with the native *Pp*HpdR/P_*hpdH*_ switchable system, pEH008. The compounds were supplemented at a concentration of 5 mM, except for DL-2-hydroxybutyrate (**20**) which, due to toxic effects, was used at 2.5 mM in *C. necator*. The response of the reporter system to the different molecules is illustrated as induction level relative to the one which was achieved by adding 5 mM 3-HP to the culture (Fig. 4b).

In addition to 3-HP, the monocarboxylic acids propionate (**12**) and butyrate (**13**), along with the structurally very similar D-3-hydroxybutyrate (D-3-HB, **21**) and L-3-hydroxybutyrate (L-3-HB, **22**), demonstrate statistically significant (p < 0.01) induction of reporter gene expression in both *E. coli* and *C. necator*. The highest level of induction relative to 3-HP was achieved with the D-enantiomer of 3-HB (**21**) in *E. coli*. It was able to induce RFP expression almost to one third of the level that had been obtained with 3-HP. The fact that in *E. coli* the L-enantiomer of 3-HB (**22**) only induces to half of the level of D-3-HB (**21**) indicates a stereospecific preference of HpdR for the D-enantiomer.

Whereas the relative induction levels for propionate (**12**) and butyrate (**13**) are roughly the same in both microorganisms when RFP expression under uninduced conditions is subtracted, a similar correlation cannot be observed for the 3-HB enantiomers. The relative induction mediated by D-3-HB (**21**) is 1% in *C. necator* as opposed to 28% in *E. coli*, whereas the L-enantiomer demonstrates a higher relative induction in *Cupriavidus*. This antagonistic behaviour in both investigated microorganisms was hypothesized to be caused by rapid metabolism of the natural D-enantiomer in *C. necator*. It encodes two D-3-hydroxybutyrate dehydrogenases (E.C. 1.1.1.30), which can convert D-3-HB (**21**) into acetoacetate. *E. coli* MG1655 completely lacks these enzymes. To test this hypothesis, *C. necator* and *E. coli* were cultivated in the presence of D- or L-3-HB (**21**, **22**, respectively). Consumption of these compounds was monitored by HPLC analysis of cell-free supernatant samples.

As hypothesized, the concentration of D-3-HB (**21**) in the supernatant of the *C. necator* culture decreased rapidly (Fig. 4c). From the initial concentration of 5 mM D-3-HB (**21**), only 0.3 ± 0.1 mM remained in the culture supernatant three hours after it had been added. Surprisingly, the non-natural L-enantiomer was consumed as well. Its concentration does not decrease as quickly as D-3-HB (**21**), however, even L-3-HB (**22**) is fully depleted six hours after its supplementation. *E. coli* on the other hand consumes neither of the 3-HB enantiomers. Varying consumption rates of D- and L-3-HB (**21**, **22**, respectively) in *C. necator* may explain the discrepancy between the relative induction levels of both microorganisms. In *C. necator*, the rate of D-3-HB (**21**) consumption appears to be higher than the rate of TIM-regulator-DNA complex formation. This subsequently results in decreased gene transcription. Even though it is metabolized, the concentration of L-3-HB (**22**) remains still high enough over the period of cultivation to mediate transient reporter gene expression.

### 3-HP-inducible system kinetics and dynamics

**Figure 5:**
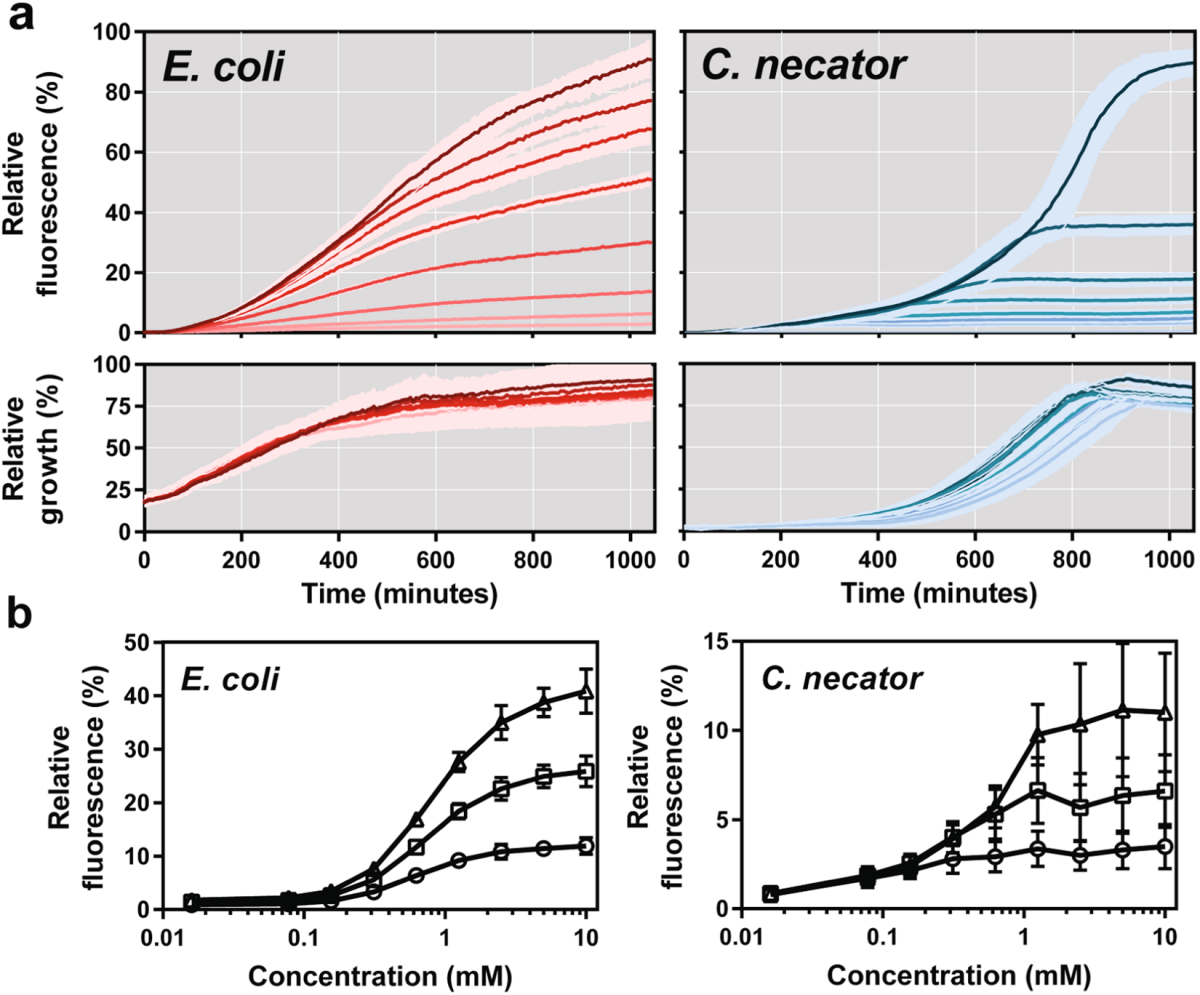
Induction dynamics and kinetics of the *Pp*HpdR/P_*hpdH*_ switchable system. (**a**) Relative fluorescence and absorbance curves of *E. coli* MG1655 and *C. necator* H16 cultures harbouring the native *Pp*HpdR/P_*hpdH*_ switchable system, pEH008. 3-HP was supplemented at time zero to the final concentrations of 10, 5, 2.5, 1.25, 0.625, 0.156, 0.016 mM and no inducer. The darker the shade of colour, the higher the level of 3-HP. The standard error of three biological replicates is illustrated as band. (**b**) Dose response curve of the *Pp*HpdR/P_*hpdH*_ switchable system in *E. coli* MG1655 and *C. necator* H16. It illustrates the relation between inducer concentration and fluorescence output 4 (circle), 6 (square) and 8 (triangle) hours after 3-HP addition. Inducer levels range from 0, 0.016, 0.078, 0.156, 0.313, 0.625, 1.25, 2.5, 5 and 10 mM. Error bars represent standard deviations of three biological replicates.

The *Pp*HpdR/P_*hpdH*_ switchable system was analysed for fluorescence output over time at different concentrations of 3-HP. This time course experiment was performed in both microorganisms and provides information about the time that is required to activate reporter expression, the influence of different inducer levels on growth and the dynamic range^32, 50^. In both microorganisms, RFP expression above the level of the uninduced culture started 30 minutes after addition of 3-HP (Fig. 5a, Supplementary Table S3). However, the profile of induction kinetics differs strongly in *E. coli* and *C. necator*. The variability can be explained by their growth kinetics and their dose response. In *E. coli*, growth is not affected by the 3-HP concentrations that were tested. In *C. necator*, however, the growth profile changes as a result of increase in 3-HP concentration. The addition of 10 mM 3-HP has a growth-retarding effect at the beginning of cultivation. The stationary growth phase is reached earlier in the presence of higher than with lower inducer concentrations, most likely due to an increased 3-HP metabolism as it has been reported for *P. denitrificans* ^10^ and other species that possess *mmsR-mmsA-hbdH* and *hpdR-hphH* regulons^24^. The metabolism of inducer in *C. necator* in turn results in transient gene expression and only higher levels of 3-HP are able to maintain a fluorescence output over the time course of experiment. This behaviour is reflected in the dose response curve for *C. necator*, which illustrates the correlation between inducer concentration and fluorescence output 4, 6 and 8 hours after 3-HP addition (Fig. 5b). Since 3-HP is not metabolized in *E. coli*, a sustained RFP expression can be observed throughout the time course of experiment. As it can be seen in the dose response curve, HpdR demonstrates a high induction cooperativity and gene expression can be tuned precisely in the range of 0.1–3 mM for a linear fluorescence output.

## Discussion

Biosustainable production of chemicals and fuels is becoming increasingly important in the global need to reduce air pollution and greenhouse gas emissions. 3-HP is an important platform chemical used as a precursor for the synthesis of acrylic acid and production of other value-added compounds. Recently, substantial metabolic engineering efforts have been made to develop microbial chassis and biosynthetic pathways for the biological production of 3-HP.

TR-based switchable systems are suitable for controlling metabolic pathways and screening microorganisms with improved production of target compounds. They have been shown to be indispensable in the iterative design–build–test cycles of synthetic biology and biotechnology, and can lead eventually to the development of improved and sustainable biosynthesis processes^31, 35^. In this study, we identified potential 3-HP-inducible systems in *P. putida* KT2440 and *C. necator* H16, each encoding two regulons putatively involved in 3-HP metabolism. They were analysed for functionality in the well-characterised microorganism *E. coli* and the chemolithoautotrophic bacterium *C. necator* which is of industrial interest due to its ability to utilise CO_2_ gas as sole carbon source.

The *mmsR-mmsA-hbdH* and *hpdR-hphH* regulons are widespread amongst proteobacteria and actinobacteria as shown previously^24^. In this study, we established that neither the *PpmmsA* nor *PphpdH* promoter can be induced by the chromosomally encoded *Cn*HpdR in the absence of *P. putida* MmsR or HpdR homologues, respectively. This suggests that MmsR and HpdR homologues and/or their respective *mmsA* and *hpdH* promoter regions in *C. necator* (betaproteobacteria) and *P. putida* (gammaproteobacteria) have diverged to the extent that their corresponding species-specific LTTRs are required for transcription activation. These findings indicate that the *Pp*MmsR/P_*mmsA*_ and *Pp*HpdR/P_*hpdH*_ switchable systems can be utilised for TR-dependent orthogonal gene expression control in biotechnologically relevant microorganisms from two different bacterial classes, gammaproteobacterium *E. coli* and betaproteobacterium *C. necator*. It was observed that absolute reporter gene expression from both *P. putida* switchable systems is much higher in *C. necator* than in *E. coli*, which results in overall higher induction levels in *Cupriavidus*. This behaviour may be due to a better expression of the *P. putida* transcriptional regulators in *C. necator*. The GC content of the TR coding sequences (62% and 64% for *Pp*MmsR and *Pp*HpdR, respectively) is more similar to the median whole genome GC content of *C. necator* (66.3%) when compared to *E. coli* (50.6%). Codon-optimisation of the regulator coding sequences for *E. coli* may result in higher absolute reporter gene expression in this microorganism. Since *Pp*HpdR/P_*hpdH*_ showed the highest induction level of the analysed 3-HP-inducible systems (516.6-fold induction in *C. necator*) and is controlled independently from host-originating TRs in both analysed organisms, it was subjected to further characterisation.

Although in this study our aim was to uncover an orthogonal 3-HP-inducible system that can be utilised for synthetic biology and biotechnology applications, a significant attempt was made to characterise the molecular mechanisms involved in *Pp*P_*hpdH*_ activation and *Pp*HpdR-P_*hpdH*_ interaction. A thorough understanding of these mechanisms is crucial for genetic part design in order to build synthetic metabolic pathways for the production of added-value compounds such as 3-HP. The bioinformatically identified conserved 40-nucleotide sequence within P_*hpdH*_ and the *in vivo* analysis of various promoter mutations suggests, that two proposed *cis*-acting regulatory elements are potentially required for promoter region interaction with HpdR to mediate transcriptional activation. The length, location and the motif complexity of the TR binding site is common for almost all LysR-type inducible promoters reported so far^26^. All *Pseudomonas* LTTRs have been clustered into 9 phylogenetic groups on the basis of full length and domain sequence analysis^29^. Interestingly, HpdR belongs to group VI, which includes only a few LysR-type proteins. None of the regulators or their potential target DNA-binding sites have been characterised to date. The novelty of the motif identified in this study can be further highlighted by the observation that neither CRE1 nor CRE2 contains the typical LysR T-N_11_-A binding motif. Furthermore, the two motifs T-N_11_-A and TTA-N_7/8_-GAA which were proposed by Zhou *et al*.^24^ to be the HpdR regulatory binding site in *P. denitrificans* ATCC 13867 could neither be detected in the *P. putida hpdH* promoter upstream of the predicted −35 box, nor do these motifs seem to be conserved across the analysed *Pseudomonas* species. It should be noted that we confirmed *in vitro* binding of HpdR to the native *hpdH* promoter region by EMSA. Protein-DNA complex formation occurs even under uninduced conditions likely indicating that HpdR is involved in negative autoregulation of *hpdR* transcription. The mechanism of negative autoregulation is typical for LTTRs^25^. Interestingly, a stronger retardation effect of protein-DNA complex was observed in the presence of 3-HP. Ligand binding may facilitate formation of tighter and higher order protein-DNA complexes required for transcription activation as reported for *catBC* promoter^48^.

In addition to characterisation of molecular mechanisms involved in promoter activation, we determined the ligand-specificity of the system as well as induction kinetics and dynamics. The analysis of structurally similar compounds revealed a set of ligand features that are necessary to interact with HpdR. The minimum requirement for the ligand to be functional is one carboxyl group, as in propionate (**12**) and butyrate (**13**). The presence of a hydroxyl group at the β-position enhances regulator activity. However, replacing the hydroxyl group by an amine- or a carboxyl group, changing the position of the hydroxyl group from β to α, or adding more functional groups, renders the ligand inactive. Cross-induction by these compounds appears to be caused by a lack of specificity for HpdR, rather than by their conversion into the primary inducer 3-HP. In this context, we uncovered the structurally similar 3-HB to be a metabolically inert analogue inducer in *E. coli*. However, due to their metabolism in *C. necator*, none of the compounds that were able to activate the *Pp*HpdR/P_*hpdH*_ switchable system can be applied as 3-HP analogue inducer for control of sustained gene expression. Metabolism and growth-retarding effects of the primary inducer were demonstrated to impact induction and growth kinetics in *C. necator*. Finally, we propose that the *Pp*HpdR/P_*hpdH*_ switchable system can be a useful genetic tool to: (i) build a 3-HP or 3-HB biosensor which reports intracellular metabolite concentrations by fluorescence output, (ii) implement directed-evolution strategies for high-throughput screening of strains with improved production titres, and (iii) to balance enzyme levels in metabolic pathways that accumulate these compounds as final product or utilise them as intermediate compound.

## Methods

### Bacterial strains, medium and growth conditions

*E. coli* TOP10 (Invitrogen, Carlsbad, CA, USA) was used for cloning and plasmid propagation. RFP fluorescence assays were performed in wild type *E. coli* MG1655^51^ and *C. necator* H16 (ATCC 17669). Bacterial strains were propagated in lysogeny broth (LB). For reporter gene assays, *E. coli* MG1655 was grown in M9 minimal growth medium supplemented with 1 µg/ml thiamine, 20 µg/ml uracil^52^ and 0.4% (w/v) glucose. *C. necator* reporter gene assays were performed in minimal medium containing 0.4% (w/v) sodium gluconate as carbon source^53^. If appropriate, antibiotics were added to the growth medium at the following concentrations: 25 µg/ml chloramphenicol or 50 µg/ml kanamycin for *E. coli* and 50 µg/ml chloramphenicol for *C. necator*. *E. coli* TOP10 was grown at 37 °C. *C. necator* H16 was cultivated at 30 °C. For comparison, both *E. coli* MG1655 and *C. necator* H16 RFP fluorescence assays were performed at 30 °C.

### Cloning and transformation

Plasmid minipreps were carried out using the New England BioLabs miniprep kit (NEB, Ipswich, MA, USA). Microbial genomic DNA was extracted employing the SigmaElute^*TM*^ bacterial genomic DNA kit (Sigma, St. Louis, MO, USA). For cloning, DNA was amplified by PCR using Phusion High-Fidelity DNA polymerase from New England BioLabs in 50 µl reactions under recommended conditions. Restriction enzymes and NEBuilder Hifi DNA assembly master mix were purchased from NEB and reactions were set up according to the manufacturers’ protocol. The Zymoclean^*TM*^ gel DNA recovery kit (Zymo, Irvine, CA, USA) was used to extract gel purified linearized DNA which was subsequently used for cloning.

Chemical competent *E. coli* were prepared and transformed by heat shock as previously described^54^. Electrocompetent *C. necator* were prepared and transformed as reported by Ausubel *et al*.^55^.

### Plasmid construction

Oligonucleotide primers were synthesized by Eurofins Genomics (Ebersberg, Germany). Primer sequences are listed in Supplementary Table S4. Plasmids were constructed using either the NEBuilder Hifi DNA assembly method according to the manufacturers’ protocol or by conventional restriction-based cloning procedures^54^. Based on the modular vector system which has been developed for the genus *Clostridium* ^42^, a modular *C. necator*-*E. coli* shuttle vector was designed. All plasmids contain a chloramphenicol resistance gene flanked by PmeI and FseI restriction sites, a pBBR1 origin of replication flanked by SbfI and PmeI^41^ and an application-specific module flanked by SbfI and AscI restriction recognition sites.

The application-specific module contains the switchable system and a *rfp* reporter gene. The coding sequences for the transcriptional regulator and RFP are arranged in opposite direction. To prevent transcriptional read-through, transcription of the regulator and the reporter gene is terminated by a *rrnB1* and a double terminator, respectively^44, 56^. Promoter and regulator sequences were verified by DNA sequencing (Source BioScience, Nottingham, UK). Plasmids used and generated in this study are listed in Supplementary Table S5. The nucleotide sequence of plasmids pEH006, pEH007, pEH008, pEH009, and pEH010 have been deposited in the public version of the JBEI registry (https://public-registry.jbei.org) under the accession numbers JPUB_008750, JPUB_008751, JPUB_008752, JPUB_008753, and JPUB_008754, respectively.

In order to compare gene expression in response to 3-HP to frequently applied switchable systems, a modular broad-host range vector containing the arabinose-inducible gene expression system was assembled. This vector was subsequently employed to generate all other constructs. pEH006 was assembled by using the NEBuilder Hifi DNA assembly method. Oligonucleotide primers EH001_f and EH002_r, EH003_f and EH004_r, EH005_f and EH006_r were used to amplify the replication origin and the chloramphenicol resistance gene, the *rfp* reporter gene and the arabinose-inducible system from pBBR1MCS-2-PphaC-eyfp-c1 and pKTrfp, respectively^44, 57^. The primer overhangs were designed to contain PmeI, FseI, AscI and SbfI restriction sites to allow for modular assembly and AatII and NdeI sites to be able to replace the switchable system. Since the transcriptional start site (+1) from the arabinose inducible promoter was known, the sequence downstream of +1 was replaced with a T7 mRNA stem-loop and a ribosome binding site from *E. coli* to improve mRNA stability and ribosome binding^44^.

pEH006E contains the promoter-less *rfp* reporter gene. This plasmid was employed to evaluate the impact of transcriptional read-through from the vector backbone on reporter gene expression. The *rfp* reporter gene was amplified with oligonucleotide primers EH013_f and EH148_r from pKTrfp^44^ and cloned into pEH006 by AatII and SbfI restriction sites.

The putative 3-HP-inducible systems *Pp*MmsR/P_*mmsA*_, *Pp*HpdR/P_*hpdH*_, *Cn*AraC/P_*acaD*_ and *Cn*HpdR/P_*mmsA1*_ were amplified with oligonucleotide primers EH017_f and EH018_r, EH019_f and EH020_r, EH021_f and EH022_r, EH023_f and EH024_r, respectively, from *P. putida* KT2440 (*Pp*) and *C. necator* H16 (*Cn*) genomic DNA and cloned into pEH006 by AatII and NdeI restriction sites. The 3-HP-inducible promoters *Pp*P_*mmsA*_, *Pp*P_*hpdH*_ and *Cn*P_*mmsA1*_ were amplified with oligonucleotide primers EH096_f and EH095_r, EH059_f and EH060_r, EH098_f and EH097_r, respectively, from *P. putida* KT2440 and *C. necator* H16 genomic DNA and cloned into pEH006 by AatII and NdeI restriction sites.

*Pp*P_*hpdH*_ promoter truncations and mutations are based on vector pEH036 (*hpdh*-118::*rfp*) which was assembled by using the NEBuilder Hifi DNA assembly method. Oligonucleotide primers EH061_f and EH062_r, EH099_f and EH100_r were used to generate a plasmid identical to pEH008; however, the bioinformatically identified mRNA stem loop structure within the *hpdH* promoter was replaced by an AvrII restriction site to allow further promoter modifications. The truncated *hpdH* promoter version *hpdh*-106::*rfp* was amplified with oligonucleotide primers EH138_f and EH139_r from pEH008 and cloned into pEH036 by AvrII and NdeI. Promoter mutations were incorporated by amplifying the promoter region from pEH008 with oligonucleotide primers that contain the desired mutation in their overhang. Generally, A’s were replaced by G’s, T’s were replaced by C’s and vice versa. Promoter versions *hpdh*-118::*rfp*_mut1 – mut12 were constructed with reverse primers EH178, EH165 – EH174, EH179, respectively, and forward primer EH175. The PCR product was cloned into pEH036 by AvrII and NdeI.

### RFP fluorescence assay

For evaluation of RFP fluorescence at a single time point, single colonies of freshly transformed *E. coli* MG1655 or *C. necator* H16 were used to inoculate 2 ml of minimal medium in 30 ml culture tubes. Cells were grown for 18 or 24 hours, respectively, before being diluted 1:20 into 2 ml fresh minimal medium. After the stock inducer was added at an OD_600_ of 0.5, cultures were cultivated for six hours at 30 °C and 200 rpm. Subsequently, 100 µl of cells were transferred to a 96-well clear-bottom plate (Greiner Bio One International, Germany; Cat. no 655090) and fluorescence was measured using an Infinite® M1000 PRO (Tecan, Switzerland) micro plate reader with 585 nm as excitation wavelength and an emission wavelength of 620 nm. The gain factor for fluorescence was set manually to 100%. Additionally, the OD_600_ was determined to normalize fluorescence by optical density.

For the growth and fluorescence time course experiments, *E. coli* MG1655 and *C. necator* H16 precultures were prepared as for the single time point measurements. They were diluted 1:40 into fresh minimal medium and incubated at 200 rpm and 30 °C until an OD_600_ of 0.2 was reached. 142.5 µl of the log-phase cells were transferred to a well of a 96-well plate. To the cultures, 7.5 µl fresh minimal medium were added containing the stock inducer at the desired concentration. Fluorescence and absorbance were measured as described earlier using Tecan micro plate reader every 5 min for 16 hours.

To determine the background autofluorescence, a culture was included in a separate well containing a control strain transformed with the empty plasmid pEH006E. Prior calculation, fluorescence and absorbance values were corrected by medium fluorescence and absorbance. Normalized fluorescence values for each measurement were obtained by dividing fluorescence by absorbance and subtracting the normalized background autofluorescence. Induction factors represent the ratio of normalized fluorescence of induced cells to normalized fluorescence of uninduced cells. Relative induction levels (%) are calculated by dividing the normalized fluorescence of cells treated with an inducer by the normalized fluorescence of cells that were induced with 3-HP.

### Metabolite consumption assay

To analyse the consumption of 3-HB by *E. coli* MG1655 and *C. necator* H16, single colonies of the wild type strains were used to inoculate 2 ml of minimal medium. From the saturated overnight culture, 6 ml of fresh medium were inoculated 1:100 in 50 ml Falcon tubes and incubated at 30 °C and 200 rpm. At an OD_600_ of 0.4–0.5, stock solutions of D- (**21**) or L-3-HB (**22**) were added to the cultures to a final concentration of 5 mM. 0.5 ml samples were taken immediately, 1, 2, 3, 6 and 24 hours after supplementation of 3-HB. The OD_600_ was determined for every time point. Subsequently, samples were centrifuged for 5 min and 13,000 rpm. The supernatant was removed and subjected to HPLC analysis as described previously^58^ with slight modifications. Briefly, to the cell-free supernatant samples, an equal volume of mobile phase was added which was spiked with 50 mM valerate as internal standard. The mobile phase was composed of 0.005 M H_2_SO_4_. Subsequently, the mixture was passed through a 0.22 µm pore size membrane filter. Samples were analysed using a Thermo Scientific Ultimate 3000 HPLC system equipped with a Phenomenex Rezex ROA-organic acid H+ (8%) 150 mm × 7.8 mm × 8 µm column and a diode array detector with the wavelength set at 210 nm. The column was operated at 35 °C with an isocratic flow rate of 0.5 ml/min. Samples were run for 30 min and the injection volume was 20 µl.

### Determination of consensus sequence

The nucleotide sequences of all retrieved *hpdR*/*hpdH* intergenic regions were aligned by using Clustal Omega^59, 60^. Subsequently, a sequence similarity motif was generated with WebLogo^61^. Putative RNA polymerase binding sites and the *hpdH* transcriptional start site were predicted by using the programs BPROM and NNPP, respectively^62, 63^.

## Electronic supplementary material

Supplementary information

Supplementary Table S3
