# Supplementary material for: Characterisation of a 3-hydroxypropionic acid-inducible system from Pseudomonas putida for orthogonal gene expression control in Escherichia coli and Cupriavidus necator
Source: Sci Rep. 2017 May 11;7:1724. doi: 10.1038/s41598-017-01850-w (PMC5431877; doi:10.1038/s41598-017-01850-w)
Supplement: Supplementary file 1 — Supplementary information [file 41598_2017_1850_MOESM1_ESM.pdf]

Supplementary information

Characterisation of a 3-hydroxypropionic acid-inducible system from *Pseudomonas putida* for orthogonal gene expression control in *Escherichia coli* and *Cupriavidus necator*

Erik K. R. Hanko[1], Nigel P. Minton[1], Naglis Malys[1]*

[1]BBSRC/EPSCR Synthetic Biology Research Centre (SBRC), School of Life Sciences, Centre for Biomolecular Sciences, The University of Nottingham, Nottingham NG7 2RD, United Kingdom

*Author to whom correspondence should be addressed, E-Mail: Naglis.Malys@nottingham.ac.uk

Supplementary Methods

Expression and purification of HpdR

Multiple attempts to purify the transcriptional regulator HpdR by His- or Strep-tag affinity chromatography resulted in insoluble protein, confirming previous observations that a majority of members from the LysR family tend to readily form inclusion bodies[1]. However, a small amount of soluble protein was obtained as a glutathione S-transferase (GST)-tagged version of HpdR. The vector encoding GST-HpdR fusion protein, pEH089, was assembled by NEBuilder Hifi of *hpdR*, which was amplified with oligonucleotide primers EH196_*r* and EH197_*f*, and BamHI/NotI digested pGEX-6P-1. To decidedly ensure that potential DNA-shifts are caused by HpdR binding, GST was simultaneously expressed from the original vector that was employed to construct the GST-HpdR fusion, pGEX-6P-1 (GE Healthcare), purified and subjected to electrophoretic mobility shift assay.

100 ml of fresh LB-ampicillin (100 µg/ml) were inoculated 1:100 with an overnight culture of *E. coli* Rosetta (DE3) (Novagen) harbouring either pGEX-6P-1 or pEH089. The cultures were incubated at 37°C and 200 rpm. At an OD_{600} of 0.5, protein expression was induced with 0.5 mM isopropyl-β-D-thiogalactopyranoside (IPTG). The cultures were grown for another 3 hours at 20°C and 220 rpm. Subsequently, cells were harvested by centrifugation at 6,000 rpm and 4°C for 8 minutes, resuspended in 2 ml phosphate buffered saline (PBS) and centrifuged as before. The supernatant was removed and pellets were frozen at -20°C.

Thawed cells were resuspended in 1 ml lysis buffer (50 mM HEPES pH 7.5, 300 mM NaCl, 10 mM $MgSO_4$, 0.2% NP-40, 5 µl protease inhibitor cocktail III (Calbiochem), 10 ng DNaseI) and lysed by sonication (10 cycles à 10 micron, 30 seconds on ice in between cycles). The cell lysate was cleared by centrifugation at 21,130 x g and 4°C for 30 minutes. The supernatant was transferred to 100 µl glutathione sepharose 4B beads (GE Healthcare) which had been equilibrated three times with 0.5 ml washing buffer (50 mM HEPES pH 7.5, 150 mM NaCl, 0.2% NP-40). The suspension was incubated at 150 rpm and 4°C for 2 hours on a horizontal shaker. Subsequently, the sepharose beads were centrifuged at 500 x g at 4°C for 5 minutes and washed five times with washing buffer. The supernatant was removed, beads were resuspended in 100 µl elution buffer A (50 mM HEPES pH 7.9, 10 mM reduced glutathione, 0.2% NP-40) and incubated at 100 rpm and 4°C for 1 hour. To the suspension, 100 µl of protease cleavage buffer (50 mM HEPES pH 7.9, 300 mM NaCl, 2 mM EDTA, 2 mM DTT, 0.2% NP-40) were added together with 12 units of PreScission Protease (protease-GST fusion, GE healthcare) and mixed at

100 rpm and 4°C for another 16 hours. The PreScission Protease-cleaved and in the supernatant released proteins (elution fraction A) were separated from the beads by centrifugation at 500 x g and 4°C for 5 minutes. Remaining unbound proteins were collected by washing twice with 150 µl elution buffer B (50 mM HEPES pH 7.9, 150 mM NaCl, 1 mM EDTA, 1mM DTT, 0.2% NP-40). Protein samples derived from the pGEX-6P-1 and pEH089 cultures were analysed by NuPAGE (4-12% Bis-Tris gel in MES running buffer, Invitrogen) according to the manufacturers' protocol.

Electrophoretic mobility shift assay (EMSA)

The purified proteins from the elution fraction A were analysed for specific binding to the native *hpdR*/*hpdH* intergenic region. Prior EMSA, the concentration of HpdR was determined by comparison to NuPAGE of bovine serum albumin (BSA) dilution series.

The ATTO700-labelled *hpdR*/*hpdH* intergenic region was generated by PCR of pEH008 with oligonucleotide primers EHseq018_ATTO700 and EHseq003. EHseq018 is 5'-ATTO700-labelled. The resulting PCR product of 310 bp was gel-purified as described earlier. The total volume of the DNA-protein-binding reaction is 10 µl. It contains 50 pM of labelled PCR product, 10 ng/µl salmon sperm DNA, 25 mM HEPES pH 7.9, 75 mM NaCl, 0.5 mM EDTA, 0.5 mM DTT, 0.1% NP-40, 150 mM KCl, 2.5 mM $MgCl_2$. A high KCl concentration has been demonstrated to facilitate DNA-protein-complex formation[2]. Furthermore, the binding reaction may contain 300 nM HpdR (elution fraction A of pEH089 culture) or an equal volume of the elution fraction A derived from pGEX-6P-1, and 1 mM of 3-HP. Samples were incubated at 20°C for 30 minutes. 10 µl of 50% (v/v) glycerol were added to each sample before being loaded onto a non-denaturing 8% Tris-Glycine gel (Invitrogen). Electrophoresis was performed in Tris-Glycine native running buffer (Invitrogen) at 4°C for 2 hours and 120V. The gel was scanned using the Odyssey Clx infrared imaging system (Model 9120, LI-COR Biosciences) and can be seen in Supplementary Fig. S2a. Additionally, the EMSA was performed using different concentrations of HpdR (Supplementary Fig. S2b).

Supplementary Tables and Figures

Supplementary Table S1. *P. denitrificans* ATCC 13867 HpdH, MmsA and MmsB homologues. The genomes of *P. putida* KT2440 and *C. necator* H16 were searched for homologues of *P. denitrificans* ATCC 13867 HpdH, MmsA and MmsB. The amino acid sequence identity (coverage) in % is listed for the identified homologues and their divergently encoded transcriptional regulators.

Strain	Operon 1<br>HpdH	Regulator	Operon 2<br>MmsA	MmsB	Regulator
P. denitrificans ATCC 13867	100	100	100	100	100
P. putida KT2440	73 (96)	73 (96)	74 (98)	62 (99)	44 (99)
C. necator H16	60 (96)	26 (64)[a]	72 (99)[b]	66 (98)	No homology[c]

[a] A *P. denitrificans* MmsA homolog is located between the *C. necator hpdH* and its putative regulator, sharing 49% identity and 96% coverage (here termed MmsA1).
[b] Here termed MmsA2.
[c] Regulator is annotated to belong to the AraC family of transcriptional regulators.

Supplementary Table S2. *P. putida* KT2440 HpdR and HpdH homologues. List of HpdR and HpdH homologues from various Pseudomonas species sharing at least 70 % protein sequence identity with *P. putida* KT2440 HpdR and HpdH, respectively.

**Species**	**HpdR Locus Tag**	**Size (aa)**	**Coverage% (identity%)**	**HpdH Locus Tag**	**Size (aa)**	**Coverage% (identity%)**
Pseudomonas putida KT2440	PP_0055	295	100 (100)	PP_0056	550	100 (100)
Pseudomonas entomophila L48	PSEEN_RS00045	295	100 (96)	PSEEN_RS00050	549	99 (91)
Pseudomonas parafulva NBRC 16636	PPA02S_RS02005	295	100 (96)	PPA02S_RS02000	548	99 (91)
Pseudomonas plecoglossicida NBRC 103162	PPL01S_RS04640	295	100 (95)	PPL01S_RS04635	548	99 (95)
Pseudomonas monteilii GTC 10897	APH46_RS25120	295	99 (95)	APH46_RS25115	548	99 (94)
Pseudomonas mosselii SJ10	O165_RS23295	295	100 (95)	O165_RS23300	549	99 (89)
Pseudomonas alkylphenolia KL28	PSAKL28_RS00210	295	100 (94)	PSAKL28_RS00215	549	99 (83)
Pseudomonas vranovensis DSM 16006	H621_RS0106435	295	100 (94)	H621_RS0106440	549	99 (84)
Pseudomonas japonica NBRC 103040	PJA01S_RS05195	295	100 (93)	PJA01S_RS05200	549	96 (81)
Pseudomonas gingeri NCPPB 3146	PGING_RS31890	295	99 (88)	PGING_RS31885	553	96 (81)
Pseudomonas fuscovaginae IRRI 6609	PF66_RS17780	295	100 (88)	PF66_RS17775	545	97 (81)
Pseudomonas taetrolens DSM 21104	TU78_RS12205	295	99 (87)	TU78_RS12200	553	96 (79)
Pseudomonas fragi B25	O5A_RS0119340	295	99 (86)	O5A_RS0119330	553	97 (80)
Pseudomonas psychrophila HA-4	B347_RS0107760	295	99 (86)	B347_RS0107770	553	96 (80)
Pseudomonas fluorescens AU12597	AA053_RS20570	295	99 (86)	AA053_RS20565	553	96 (78)
Pseudomonas agarici NCPPB 2289	PAGAR_RS0113390	295	100 (87)	PAGAR_RS0113385	553	96 (80)
Pseudomonas helleri DSM 29165	TU84_RS23875	295	100 (85)	TU84_RS23880	553	96 (80)
Pseudomonas chlororaphis 30-84	PCHL3084_RS12165	297	100 (85)	PCHL3084_RS12160	548	96 (79)
Pseudomonas deceptionensis DSM 26521	TR67_RS22630	295	99 (86)	TR67_RS22625	553	96 (79)
Pseudomonas batumici UCM B-321	UCMB321_5547	295	100 (89)	UCMB321_5548	549	99 (80)
Pseudomonas lini ZBG1	ACS73_RS06805	294	99 (85)	ACS73_RS06800	548	96 (80)
Pseudomonas umsongensis UNC430CL58Col	N519_RS0117920	308	98 (85)	N519_RS0117915	549	99 (79)
Pseudomonas cremoricolorata ND07	LK03_RS06240	295	100 (87)	LK03_RS06245	549	99 (81)
Pseudomonas protegens Cab57	PPC_RS12670	297	99 (83)	PPC_RS12665	548	98 (77)
Pseudomonas frederiksbergensis SI8	JZ00_RS11015	294	99 (84)	JZ00_RS11010	547	97 (79)
Pseudomonas mediterranea TEIC1105	ADY55_RS24405	294	99 (84)	ADY55_RS24410	547	97 (80)
Pseudomonas brassicacearum PA1G7	AW28_RS07090	294	99 (83)	AW28_RS07085	547	95 (81)
Pseudomonas corrugata TEIC1148	ADY50_RS18415	294	99 (82)	ADY50_RS18420	547	97 (79)
Pseudomonas trivialis IHBB745	AA957_RS03805	297	100 (79)	AA957_RS03810	548	96 (78)
Pseudomonas orientalis DSM 17489	TU82_RS23530	296	100 (80)	TU82_RS23535	548	96 (77)
Pseudomonas poae DSM 14936	TU75_RS20075	298	99 (79)	TU75_RS20070	548	96 (78)
Pseudomonas tolaasii 6264	UQW_RS0110215	297	100 (79)	UQW_RS0110210	548	98 (78)
Pseudomonas veronii R4	SU91_RS20320	297	99 (79)	SU91_RS20325	555	96 (78)
Pseudomonas synxantha DSM 18928	TU77_RS19905	297	100 (79)	TU77_RS19910	548	99 (77)
Pseudomonas marginalis ICMP 9505	AO391_RS22020	297	100 (79)	AO391_RS22025	548	96 (77)
Pseudomonas rhizosphaerae IH5	LT40_RS16020	295	99 (83)	LT40_RS16015	550	98 (78)
Pseudomonas aeruginosa AZPAE14918	NS34_RS16205	301	99 (73)	NS34_RS16200	557	96 (74)
Pseudomonas denitrificans ATCC 13867	H681_RS18595	304	99 (73)	H681_RS18590	554	96 (73)
Pseudomonas knackmussii B13	PKB_RS05640	301	99 (72)	PKB_RS05645	552	97 (72)
Pseudomonas nitroreducens Aramco J	QX33_RS11735	300	99 (74)	QX33_RS11740	554	96 (73)
Pseudomonas citronellolis TTU2014-008ASC	AO742_RS16215	297	99 (70)	AO742_RS16210	552	97 (73)

Supplementary Table S4. List of primers that were used in this study. Restriction sites that were incorporated for cloning are underlined.

Primer name	Primer sequence (5'→ 3')
EH001_f	tggtgagaatccaagcttccattcaggtcgaggtggccc
EH002_r	ttatacctagggcgttcggctgcggctggcgctgggcct
EH003_f	gccgcagccgaacgccctaggtataaacgcagaaaggccca
EH004_r	tgtttctccatagggagaccacaacggtttccctctagaaataattttggaattcaaaagatcttttaagaaggagatatacatatgg
EH005_f	accgttgtggtctccctatggagaaacagtagagagttgcgataaaaagcg
EH006_r	cctcgacctgaatggaagcttggattctcaccaataaaaaacgcccgg
EH013_f	ggatgacctgcaggtataaacgcagaaaggcccacc
EH017_f	cgccatatgcgttctccttggaattgttgtc
EH018_r	tatgacgtctcagtcctgggcaaagcg
EH019_f	cgccatatgcaacctcgcgcctg
EH020_r	tatgacgtcctactcggctagcaactcgc
EH021_f	cgccatatgagatgagggtgggggtttg
EH022_r	tatgacgtctcaaccttccgccacgc
EH023_f	cgccatatggctggcttctgcaaggatg
EH024_r	tatgacgtcctaagtccggaacaccgact
EH059_f	gctactcgccatatgcaacctcgcgcctg
EH060_r	tcgttttatgacgtcgcttgtcctttatggcagttcg
EH061_f	acttttacgcaacgcataattgttgt
EH062_f	acaacaattatgcgttgcgtaaaagt
EH095_r	tcgttttatgacgtcgatgatccggtttttttgtgcgt
EH096_f	gctactcgccatatgcgttctccttggaattgttgtc
EH099_f	gcctgtacctaggaacgaactgccataaaggacaag
EH100_r	gtcctttatggcagttcgttcctaggtacaggcttgcccctgtg
EH138_f	aacacgttcccatttgaaaccttc
EH139_r	agttcgttcctaggcctgtgctaaaacgcacagc
EH148_r	tcgttttatgacgtcatggcgagtagcgaagacgtta
EH165_r	cagttcgttcctaggcgtgggcttgcccctgtgctaaaa
EH166_r	cagttcgttcctaggtacaaatccgcccctgtgctaaaacgcac
EH167_r	cagttcgttcctaggtacaggcttatttttgtgctaaaacgcacagcgg
EH168_r	cagttcgttcctaggtacaggcttgcccccacatcaaaacgcacagcggctgcg
EH169_r	cagttcgttcctaggtacaggcttgcccctgtgctggggtgcacagcggctgcgcgaaat
EH170_r	cagttcgttcctaggtacaggcttgcccctgtgctaaaacatgtggcggctgcgcgaaatctcgt
EH171_r	cagttcgttcctaggtacaggcttgcccctgtgctaaaacgcacaataattgcgcgaaatctcgtgtttc
EH172_r	cagttcgttcctaggtacaggcttgcccctgtgctaaaacgcacagcggccatataaaatctcgtgtttcatccacgaaat
EH173_r	cagttcgttcctaggtacaggcttgcccctgtgctaaaacgcacagcggctgcgcggggcttcgtgtttcatccacgaaattactcac
EH174_r	cagttcgttcctaggtacaggcttgcccctgtgctaaaacgcacagcggctgcgcgaaatcctacatttcatccacgaaattactcac taagatgga
EH175_f	ataacgtcttcgctactcgccatatgcaacctcgcgcctgttttttat
EH178_r	cagttcgttcctaggcacaggcttgcccctgtgctaa
EH179_r	cagttcgttcctaggtacaggcttgcccctgtgctaaaacgcacagcggctgcgcgaaatctcatgtttcatccacgaaattactcac taagatg
EH196_r	ttctgttccaggggcccctgggatccatgttcgactggaatgatctgc
EH197_f	gatcgtcagtcagtcacgatgcggccgcctactcggctagcaactcgc
EHseq018_ATTO700	cgcagatcattccagtcgaaca
EHseq003	catacgaactttgaaacgcatgaact

Supplementary Table S5. List of plasmids that were used and generated in this study.

Plasmid	Characteristic	Reference or source
pBBR1MCS-2-PphaC-eyfp-c1	Kan^r; broad host range vector used to amplify the origin of replication	[3]
pKTrfp	Cm^r; vector used to amplify *cmR*, P_{araC}-*araC* and P_{araBAD}-*rfp*-T_{dbl}	[4]
pGEX-6P-1	Amp^r; vector used to generate *gst-hpdR* fusion	GE Healthcare
pEH006	Cm^r; P_{araC}-*araC*-T_{rrnB1} and P_{araBAD}-T7sl-*Ec*RBS-*rfp*-T_{dbl} from pKTrfp assembled by NEBuilder	This study
pEH006E	Cm^r; empty vector; *rfp*-T_{dbl} from pKTrfp cloned into pEH006 by AatII and SbfI sites	This study
pEH007	Cm^r; P_{mmsR}-*mmsR*-T_{rrnB1} and P_{mmsA}-*rfp*-T_{dbl} from *P.putida* KT2440 genomic DNA cloned into pEH006 by AatII and NdeI sites	This study
pEH008	Cm^r; P_{hpdR}-*hpdR*-T_{rrnB1} and P_{hpdH}-*rfp*-T_{dbl} from *P.putida* KT2440 genomic DNA cloned into pEH006 by AatII and NdeI sites	This study
pEH009	Cm^r; P_{araC}-*araC*-T_{rrnB1} and P_{mmsA2}-*rfp*-T_{dbl} from *C. necator* H16 genomic DNA cloned into pEH006 by AatII and NdeI sites	This study
pEH010	Cm^r; P_{hpdR}-*hpdR*-T_{rrnB1} and P_{mmsA1}-*rfp*-T_{dbl} from *C. necator* H16 genomic DNA cloned into pEH006 by AatII and NdeI sites	This study
pEH022	Cm^r; P_{hpdH}-*rfp*-T_{dbl} from *P.putida* KT2440 genomic DNA cloned into pEH006 by AatII and NdeI sites	This study
pEH034	Cm^r; P_{mmsA}-*rfp*-T_{dbl} from *P. putida* KT2440 genomic DNA cloned into pEH006 by AatII and NdeI sites	This study
pEH036	Cm^r; P_{hpdR}-*hpdR*-T_{rrnB1} and P_{hpdH}(-118)-*rfp*-T_{dbl} from pEH008 assembled by NEBuilder	This study
pEH053	Cm^r; P_{hpdR}-*hpdR*-T_{rrnB1} and P_{hpdH}(-106)-*rfp*-T_{dbl} from pEH008 cloned into pEH036 by AvrII and NdeI sites	This study
pEH068	Cm^r; P_{hpdR}-*hpdR*-T_{rrnB1} and P_{hpdH}(-118_mut2)-*rfp*-T_{dbl} from pEH008 cloned into pEH036 by AvrII and NdeI sites	This study
pEH069	Cm^r; P_{hpdR}-*hpdR*-T_{rrnB1} and P_{hpdH}(-118_mut3)-*rfp*-T_{dbl} from pEH008 cloned into pEH036 by AvrII and NdeI sites	This study
pEH070	Cm^r; P_{hpdR}-*hpdR*-T_{rrnB1} and P_{hpdH}(-118_mut4)-*rfp*-T_{dbl} from pEH008 cloned into pEH036 by AvrII and NdeI sites	This study
pEH071	Cm^r; P_{hpdR}-*hpdR*-T_{rrnB1} and P_{hpdH}(-118_mut5)-*rfp*-T_{dbl} from pEH008 cloned into pEH036 by AvrII and NdeI sites	This study
pEH072	Cm^r; P_{hpdR}-*hpdR*-T_{rrnB1} and P_{hpdH}(-118_mut6)-*rfp*-T_{dbl} from pEH008 cloned into pEH036 by AvrII and NdeI sites	This study
pEH073	Cm^r; P_{hpdR}-*hpdR*-T_{rrnB1} and P_{hpdH}(-118_mut7)-*rfp*-T_{dbl} from pEH008 cloned into pEH036 by AvrII and NdeI sites	This study
pEH074	Cm^r; P_{hpdR}-*hpdR*-T_{rrnB1} and P_{hpdH}(-118_mut8)-*rfp*-T_{dbl} from pEH008 cloned into pEH036 by AvrII and NdeI sites	This study
pEH075	Cm^r; P_{hpdR}-*hpdR*-T_{rrnB1} and P_{hpdH}(-118_mut9)-*rfp*-T_{dbl} from pEH008 cloned into pEH036 by AvrII and NdeI sites	This study
pEH076	Cm^r; P_{hpdR}-*hpdR*-T_{rrnB1} and P_{hpdH}(-118_mut10)-*rfp*-T_{dbl} from pEH008 cloned into pEH036 by AvrII and NdeI sites	This study
pEH077	Cm^r; P_{hpdR}-*hpdR*-T_{rrnB1} and P_{hpdH}(-118_mut11)-*rfp*-T_{dbl} from pEH008 cloned into pEH036 by AvrII and NdeI sites	This study
pEH079	Cm^r; P_{hpdR}-*hpdR*-T_{rrnB1} and P_{hpdH}(-118_mut1)-*rfp*-T_{dbl} from pEH008 cloned into pEH036 by AvrII and NdeI sites	This study
pEH080	Cm^r; P_{hpdR}-*hpdR*-T_{rrnB1} and P_{hpdH}(-118_mut12)-*rfp*-T_{dbl} from pEH008 cloned into pEH036 by AvrII and NdeI sites	This study
pEH089	Amp^r; *hpdR* from *P. putida* KT2440 genomic DNA cloned into pGEX-6P-1 by NEBuilder	This study

Supplementary Fig. S1. Secondary structure analysis of the *P. putida* KT2440 *hpdR/hpdH* intergenic region. The *hpdR/hpdH* intergenic region was analyzed with the mfold web server for potential secondary structures. Default settings were chosen to predict nucleic acid folding. The 5'- and 3'-ends represent the translational start sites of *hpdR* and *hpdH*, respectively. The calculated minimum free energy, ΔG, is -36.06 kcal/mol.

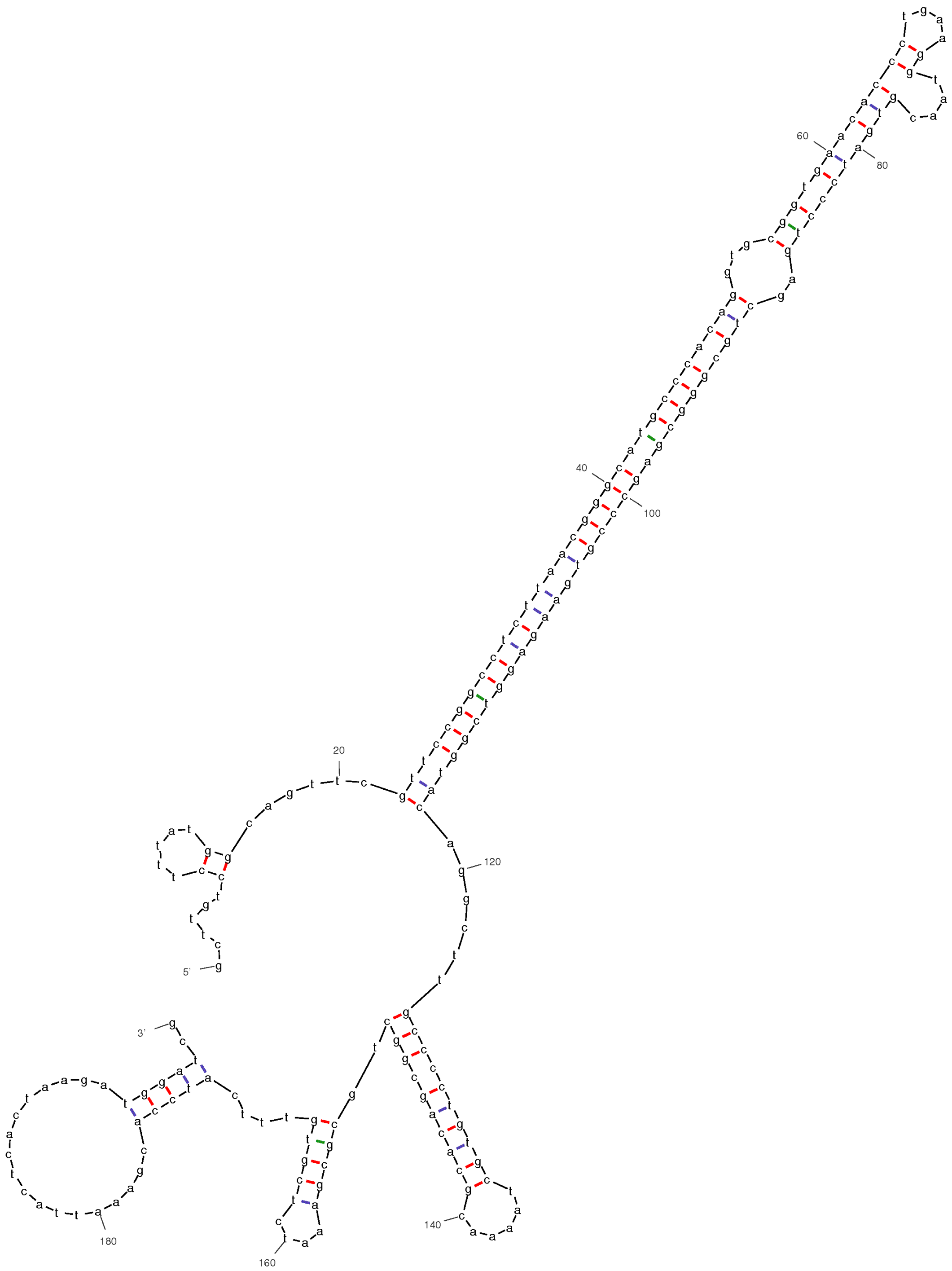

Supplementary Fig. S2. EMSA of the native *hpdR*/*hpdH* intergenic region. (**a**) Effect of purified regulator and 3-HP on migration of labelled DNA. EF(-) and EF(+) refer to elution fractions A from pGEX-6P-1 and pEH089 cultures, respectively. EF(-) does not contain the transcriptional regulator HpdR. EF(+) contains HpdR. A slower-migrating secondary DNA band can be seen in each of the lanes. Similar less abundant and slower-migrating DNA of potentially different confirmation on non-denaturing protein gels have been reported previously[5,6]. (**b**) Effect of different concentrations of purified regulator on migration of labelled DNA. HpdR concentrations are 300 nM (lane 1), 150 nM (lane 2), 75 nM (lane 3) and no regulator.

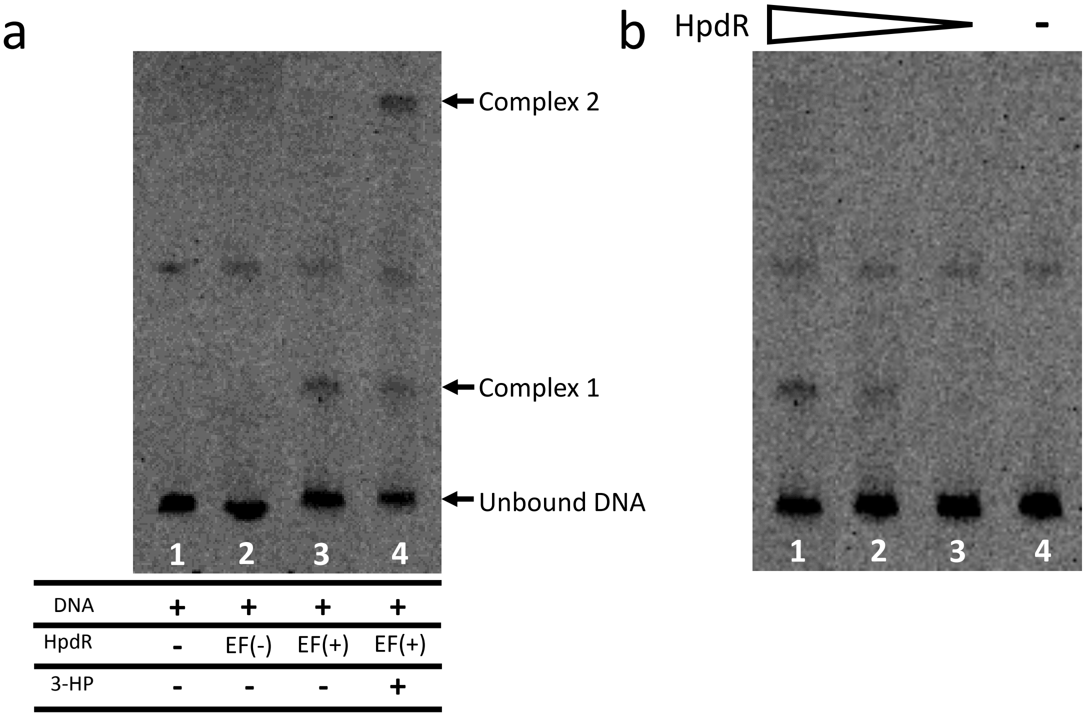

Supplementary References

1 Jørgensen, C. & Dandanell, G. Isolation and characterization of mutations in the *Escherichia coli* regulatory protein XapR. *J. Biotechnol.* **181**, 4397-4403 (1999).

2 Pande, S.G. et al. *Lactobacillus brevis* responds to flavonoids through KaeR, a LysR-type of transcriptional regulator. *Mol. Microbiol.* **81**, 1623-1639 (2011).

3 Pfeiffer, D. & Jendrossek, D. Interaction between poly(3-hydroxybutyrate) granule-associated proteins as revealed by two-hybrid analysis and identification of a new phasin in *Ralstonia eutropha* H16. *Microbiology* **157**, 2795-2807 (2011).

4 Bi, C. et al. Development of a broad-host synthetic biology toolbox for *Ralstonia eutropha* and its application to engineering hydrocarbon biofuel production. *Microb. Cell Fact.* **12** (2013).

5 Ko, M. & Park, C. H-NS-dependent regulation of flagellar synthesis is mediated by a LysR family protein. *J. Bacteriol.* **182**, 4670-4672 (2000).

6 Hoffmann, J. & Altenbucher, J. Functional characterization of the mannitol promoter of *Pseudomonas fluorescens* DSM 50106 and its application for a mannitol-inducible expression system for *Pseudomonas putida* KT2440. *PLoS ONE* **10** (2015).
